# Mechanistic insights into water vapor adsorption behavior and its impact on dynamic adsorption of acetone: experimental and molecular simulation study

**DOI:** 10.1039/d6ra06366g

**Published:** 2026-08-27

**Authors:** Lijuan Jia, Yue Guan, Yurun Tong, Yahan Cui, Tong Zhang, Kangbo Shen, Jinhao Zhang, Dayang Wu, Yiyang Guo

**Affiliations:** a Shanxi Key Laboratory of Ecological Protection and Resources Utilization of Yuncheng Salt Lake, Department of Applied Chemistry, Yuncheng University 1155 Fudan West Street Yuncheng 044000 China jialijuan1203@163.com

## Abstract

Water vapor significantly impacts volatile organic compound (VOC) adsorption in fixed-bed systems. This study elucidates water vapor's interference mechanisms during acetone adsorption. Dynamic tests (298.15–328.15 K) revealed that water breakthrough times were generally insensitive to relative humidity (RH), except at 298.15 K where duration increased with RH. A distinct plateau in the water breakthrough curve emerged at 298.15 K. This anomalous extension of water breakthrough duration at 298.15 K stemmed from enhanced water cluster formation and micropore filling under low-temperature conditions, where reduced thermal motion favored water aggregation in narrow pores. In binary systems, a bidirectional competitive displacement phenomenon occurred, referring to two concurrent processes: water displaced adsorbed acetone from high-affinity micropore sites *via* stronger hydrogen bonding, and acetone occupied pore channels, reducing pore blockage and accelerating water breakthrough. During the co-adsorption process, elevated RH amplified acetone roll-up *via* enhanced micropore-filling water clusters, while water roll-up diminished due to size-exclusion effects. Molecular simulations revealed that although water exhibited a lower average adsorption heat than acetone, it formed stable hydrogen-bonded clusters at oxygen-containing functional groups, resulting in significant localized adsorption enhancement. Although water preferentially occupied high-affinity micropore sites (∼2.7 Å) in competition with acetone, the presence of water didn't alter the primary adsorption configuration or orientation of acetone molecules. In addition, the diffusion coefficients of both components increased in the binary system, confirming that trace water promoted acetone transport *via* bidirectional competitive displacement, despite generally hindering overall adsorption.

## Abstract 2

Bidirectional competitive displacement with higher diffusion coefficient in co-adsorption system of acetone and water vapor.

## Graphical abstract

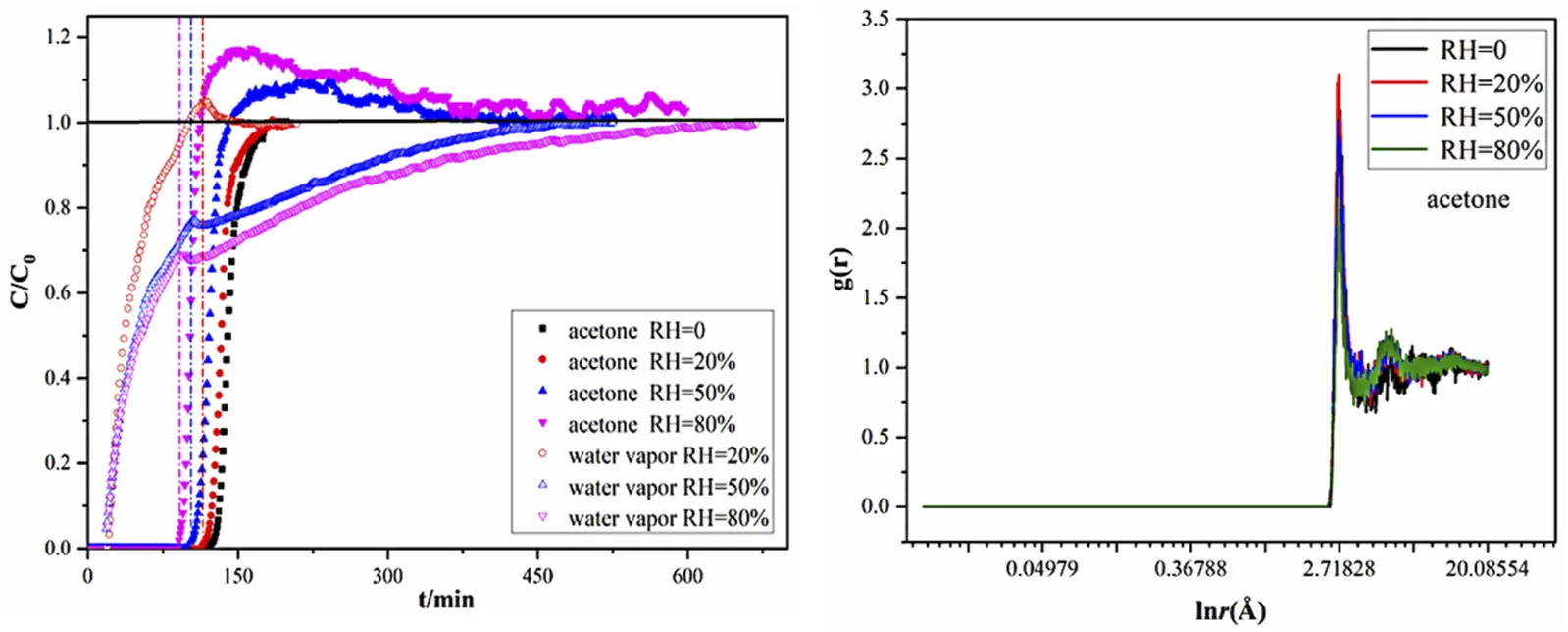

## 1 Introduction

Volatile organic compounds (VOCs) are critical precursors in tropospheric ozone formation *via* photochemical oxidation and pose significant health risks due to inherent toxicity.^1,2^ Under solar irradiation, VOCs undergo chain reactions with nitrogen oxides (NO_*x*_), catalyzing the formation of ground-level ozone and secondary organic aerosols. These key components of photochemical smog degrade atmospheric visibility and elevate respiratory health risks. Consequently, stringent VOC regulation is a pivotal strategy for air quality governance.

Adsorption technology has been extensively implemented in industrial settings for VOC abatement, serving dual functions of emission mitigation and resource recovery through retrievable VOC capture. In practical applications, however, the omnipresence of atmospheric water vapor introduces critical complexities in adsorption system performance, as it modulates both equilibrium capacities and dynamic adsorption kinetics across diverse VOC species.^3,4^ Improving the understanding of humidity effects on VOC adsorption directly supports energy-efficient VOC recovery processes. Previous studies report that optimizing adsorption conditions to mitigate humidity interference can reduce the thermal energy consumption of adsorbent regeneration, thereby lowering operational carbon emissions. This aligns with China's dual carbon goals of peaking carbon emissions by 2030 and achieving carbon neutrality by 2060. This framework recognizes that VOC control addresses both immediate air quality concerns and long-term carbon management.

Unlike equilibrium isotherms that only describe saturated adsorption capacity, dynamic breakthrough curves directly determine key fixed-bed design parameters: the breakthrough time sets the adsorbent switching/regeneration cycle, the roll-up peak amplitude dictates the downstream VOC treatment load, and the mass transfer zone length guides bed height optimization. A quantitative understanding of humidity effects on these dynamic parameters is therefore critical for designing energy-efficient VOC adsorption systems. Among various VOCs, acetone is not only widely used in industrial applications but also exhibits strikingly inconsistent responses to water vapor in adsorption processes, making it a representative and instructive case for understanding humidity effects. Shi *et al.*^5^ demonstrated that converting hydrophilic adsorbents to hydrophobic structures could mitigate the inhibitory effect of water vapor on acetone adsorption. This highlights the critical role of hydrophobic properties in co-adsorbing water vapor and volatile organic compounds (VOCs). Laskar *et al.*^6^ attributed the pronounced negative impact of water vapor on acetone adsorption to hydrogen bond formation between water and acetone molecules. Conversely, our recent investigation^7^ revealed that such hydrogen bonding mechanisms could actually counteract the adverse effects of humidity on VOC adsorption processes. Despite extensive research on humidity-dependent VOC adsorption dynamics, a universal theoretical framework capable of systematically reconciling disparate observations remains lacking.

Notably, few studies have investigated the dynamic adsorption behavior of water vapor itself—an oversight given its fundamental importance in elucidating humidity-induced inhibition mechanisms and developing effective mitigation strategies for VOC adsorption.^6,8^ As demonstrated in existing literature about water vapor adsorption,^9^ specific surface area and pore volume provide the spatial foundation for adsorption, whereas pore surface properties, including functional group types, functional group abundance, and surface roughness, govern the adsorption strength and number of adsorption sites. Kim *et al.*^10^ reported that increasing relative humidity (RH) and flow rate shortens breakthrough times and reduces mass transfer zone lengths. Ribeiro *et al.*^11^ observed two distinct breakthrough stages (favorable and unfavorable regions) for water vapor adsorption on molecular sieves. However, systematic knowledge of how key parameters—particularly temperature and RH-govern water vapor adsorption dynamics is still lacking. This knowledge gap severely limits our ability to interpret, let alone predict, the humidity-dependent behavior of VOC adsorption.

Despite extensive studies on VOC adsorption, the bidirectional competitive mechanism between water and VOCs in GAC pores lacks quantitative validation *via* combined experimental and molecular simulation approaches. To address this gap, this work integrates experimental breakthrough tests and molecular dynamics/GCMC simulations. In this work, we chose granular activated carbon (GAC) as adsorbents, which remains a widely employed adsorbent in various chemical processes, particularly for VOCs removal.^12,13^ Firstly, single-component adsorption breakthrough curve measurements for water vapor on GAC were conducted at three RH levels (20%, 50%, 80%) across four temperature points (298.15, 308.15, 318.15, and 328.15 K) using a packed bed system. Subsequently, dual-component breakthrough curves for acetone-water vapor co-adsorption were obtained at 298.15 K under identical RH conditions to systematically investigate the impact of humidity on acetone adsorption dynamics. Lastly, a hybrid simulation approach integrating Grand Canonical Monte Carlo (GCMC) and molecular dynamics (MD) was employed to elucidate the competitive adsorption mechanisms between acetone and water vapor.

## 2 Materials and methods

### 2.1 Materials

Acetone (purity >99.5%) was obtained from Nanjing Chemical Reagent Company Limited (China). The physical and chemical properties of acetone samples were systematically presented in Table S1. Granular activated carbon (GAC) employed in this study was identical to that used in our previous investigations,^14^ exhibiting characteristic micro-porous structure typical of conventional adsorbents. Detailed structural parameters and surface characteristics of GAC were provided in Table S2. Notably, the GAC demonstrated a specific surface area of 2089.3 m^2^ g^−1^ with a predominance of acidic functional groups (1.701 mmol g^−1^) on its surface. Prior to experimental use, the adsorbents were kept in a drying vacuum oven at 383.15 K for more than 2 h to remove impurities.

### 2.2 Adsorption experiments

#### 2.2.1 Dynamic adsorption of water vapor

**Fig. 1:**
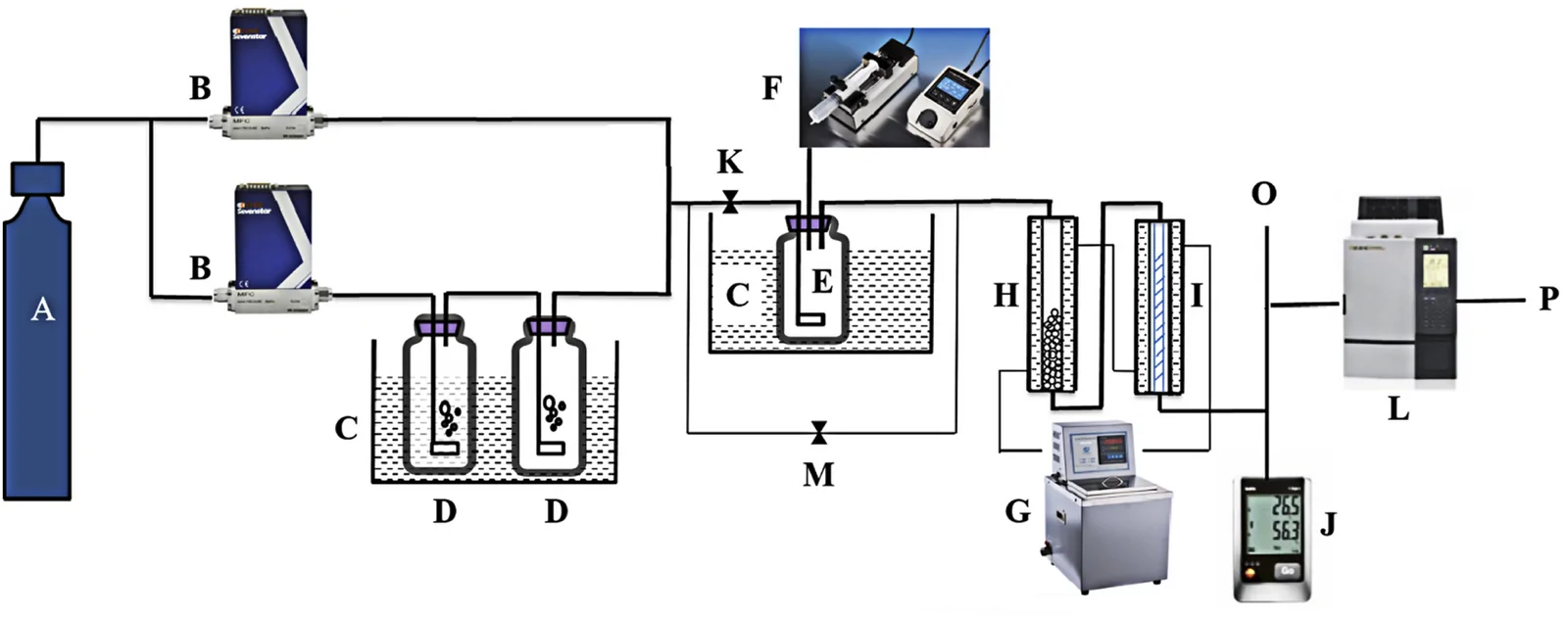
Dynamic adsorption schematic diagram of VOCs and water vapor (A). High purity nitrogen (B). Mass flow meter (C). Thermostatic water bath (D). Ultrapure water (E). Vaporizing chamber (F). Micro-injection pump (G). Circulating water bath (H). Buffer column (I). Adsorption column (J). Hygronom (L). Gas chromatograph (K). Bypass-1 (M). Bypass-2 (O). Offgas (P). Offgas).

To evaluate the adsorption dynamics of water vapor on GAC, a series of meticulously designed breakthrough experiments were conducted, and the experimental setup was schematically illustrated in Fig. 1, with bypass-1(K) closed. This comprehensive system comprised three integral components: a gas distribution subsystem, an adsorption subsystem, and a detection subsystem. The gas distribution subsystem operated as follows: high-purity nitrogen from the cylinder first traversed a dryer to remove any residual moisture. Subsequently, the airflow was bifurcated into two channels, each precisely regulated by a mass flow controller (MFC). In this experiment, the total volumetric flow rate was maintained at 100 mL min^−1^ at standard temperature and pressure (STP: 273.15 K, 101.325 kPa). To generate water vapor at different relative humidity (RH) levels of 20%, 50%, and 80%, the nitrogen gas flow was adjusted in two distinct ways. The humidified gas then passed through a glass column, measuring 10 cm in length and with an inner diameter of 1 cm, which was packed with 3.7 g of granular activated carbon (GAC). After passing through the adsorption column, the relative humidity of the effluent gas was measured using a Testo 176-H1 hygrometer (measurement range: 0–100% RH, −20 °C–70 °C, accuracy: ±0.1% RH), enabling the acquisition of water vapor breakthrough curves. The adsorption experiments were carried out at four distinct temperatures: 298.15 K, 308.15 K, 318.15 K, and 328.15 K, respectively. The actual total volumetric flow rate at four experimental temperatures according to eqn (1):

1 *V*_actual_ = *V*_STP_ × (*T*_exp_/273.15)

where *T*_exp_ is the experimental temperature in Kelvin. The actual molar flow rates at 298.15 K, 308.15 K, 318.15 K and 328.15 K are 109.10 mL min^−1^, 112.76 mL min^−1^, 116.42 mL min^−1^ and 120.08 mL min^−1^, respectively.

#### 2.2.2 Dynamic co-adsorption of acetone and water vapor

The experiment for dynamic co-adsorption of acetone and water vapor was also conducted as Fig. 1, opening bypass-1(K) and closing the bypass-2(M). Acetone and water vapor were mixed and then passed through the VOCs vaporization chamber. VOCs vaporizing chamber temperature was set at 353.15 K. By adjusting the injection pump, certain concentration (10 mg L^−1^) of acetone was obtained. Acetone (10 mg L^−1^) and water vapor (20%, 50% and 80%) were mixed together into a buffer column and the total mass flow in this experiment was also 100 mL min^−1^. Subsequently, the flow was introduced into adsorption column, maintained at 298.15 K. The detection system consisted of two parts: the hygrometer and the gas chromatograph. The hygrometer detected the humidity at the exit of the adsorption column in real time, and the gas chromatograph detected the concentration of acetone at the exit. In this study, *C*_0_ is the inlet acetone concentration stabilized before breakthrough, and *C* is the real-time outlet acetone concentration measured by the gas chromatograph. Breakthrough time (*t*_brea_) is defined as the time when the normalized outlet concentration *C*/*C*_0_ reaches 0.05, and saturation time (*t*_sat_) is defined as the time when *C*/*C*_0_ reaches 0.95. The roll-up start time is defined as *C*/*C*_0_ = 1, and the roll-up end time is defined as the time when *C*/*C*_0_ returns to 1 after the peak. These criteria are consistent with standard practices for fixed-bed breakthrough analysis and ensure full reproducibility of our results.

### 2.3 The theoretical calculation

**Fig. 2:**
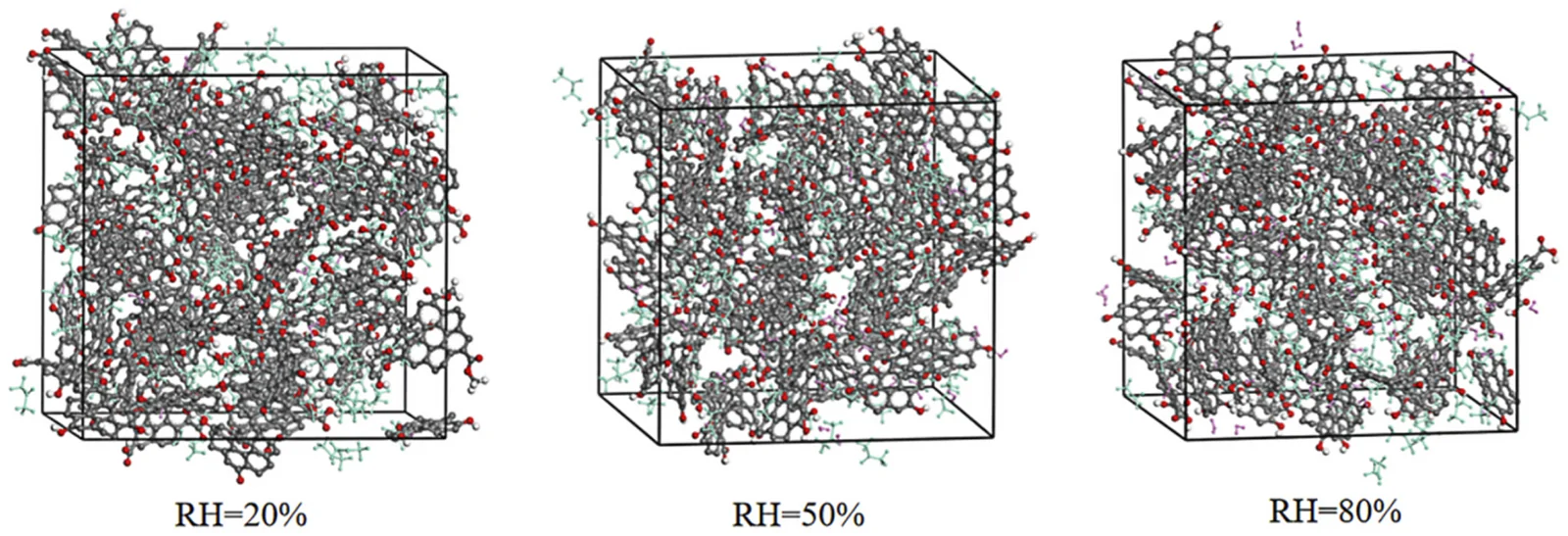
Model construction diagram for co-adsorption of acetone-water vapor on GAC.

The activated carbon model was constructed from oxygen-containing structural units, with a molar ratio of –OH : –COOH : –C=O : –COOR = 49 : 20 : 25 : 1. The simulation box size was 4.21 nm × 4.21 nm × 4.21 nm, shown in Fig. 2. Geometry optimization was performed using the DMol3 module in Materials Studio with the GGA-PBE functional. GCMC adsorption simulations were conducted using the sorption module with the Metropolis algorithm. The COMPASSIII force field was employed, the temperature was set to 298.15 K, the fugacity was 101.25 kPa, the total number of simulation steps was 1.0 × 10^6^, and the van der Waals cutoff distance was 18.5 A. Molecular dynamics simulations were performed using the Forcite Dynamics module under the NVT ensemble, with the carbon framework fixed. The simulation temperature was 298.15 K, using the Nosé thermostat to control the temperature, with a *Q* ratio value of 0.01. The time step was 1 fs, and the total simulation time was 200 ps. During the simulations, the adsorption heats, the adsorption energy, the radial distribution function (RDF) and the Mean Square Displacement (MSD) were calculated during the co-adsorption of acetone and water vapor. RDF is a function that describes the distribution of distances between different particles, which can be molecules or atoms. It reflects the changes in the density of particles in space by comparing the ratio of local density to average density.^15,16^ And MSD can be used to calculate the diffusion coefficient of acetone and H_2_O.

## 3 Results and discussion

### 3.1 Dynamic adsorption characteristic of water vapor

**Fig. 3:**
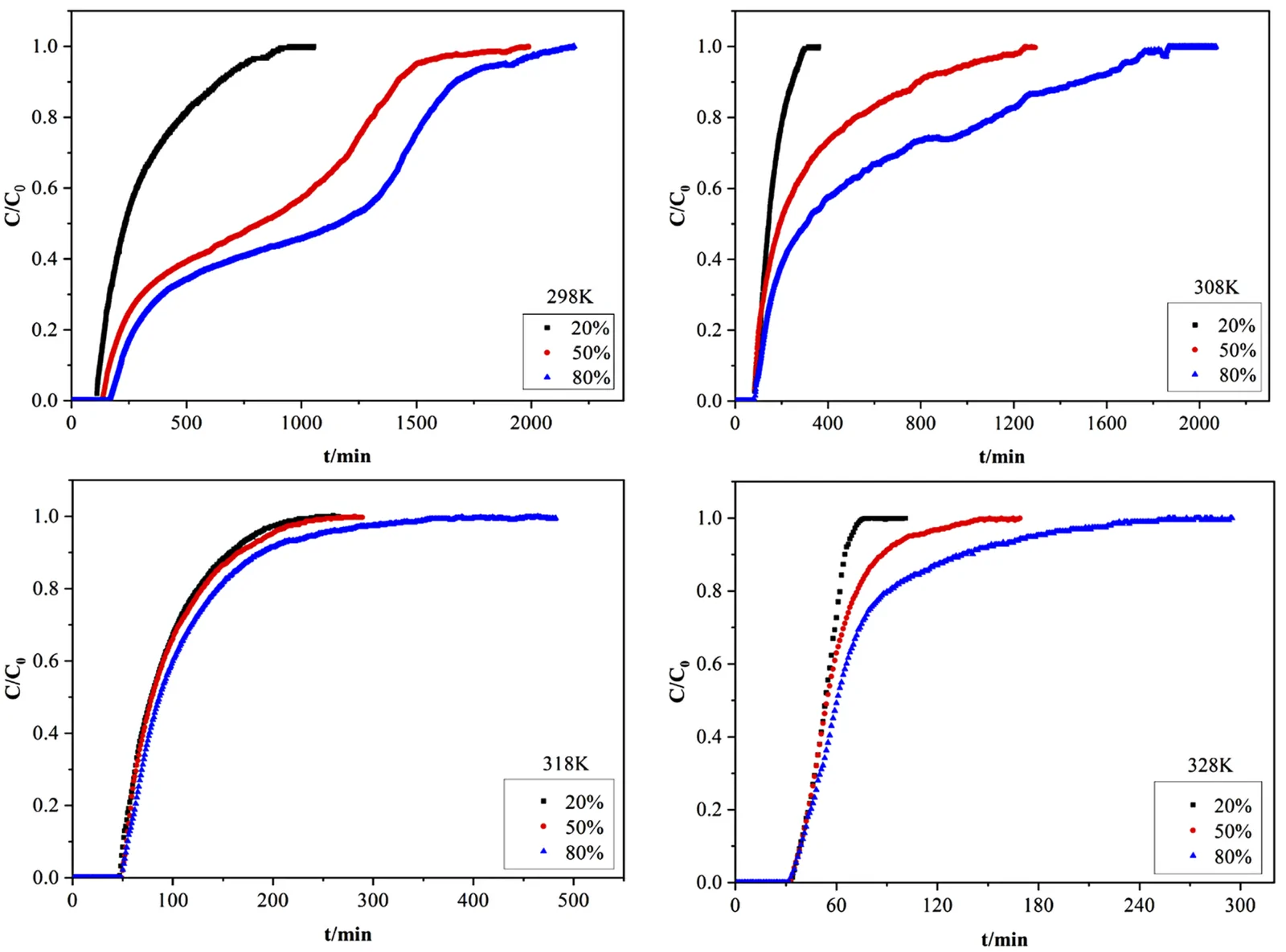
Breakthrough curves of water vapor at 298.15–328.15 K with RH of 20%, 50% and 80% adsorption on GAC.

Fig. 3 illustrated the breakthrough profiles of water vapor on microporous granular activated carbon (GAC) across three relative humidity (RH) levels (20%, 50%, and 80%) and four temperatures (298.15, 308.15, 318.15, and 328.15 K). The results indicated that the water vapor breakthrough time decreased, whereas the mass transfer rate increased with rising temperature. Notably, breakthrough time was independent of relative humidity (RH) except at 298.15 K. In contrast, the mass transfer rate exhibited a decreasing trend as RH increased. To provide deeper insight, the effects of RH and temperature on dynamic adsorption were analyzed separately below.

#### 3.1.1 Effect of relative humidity

**Fig. 4:**
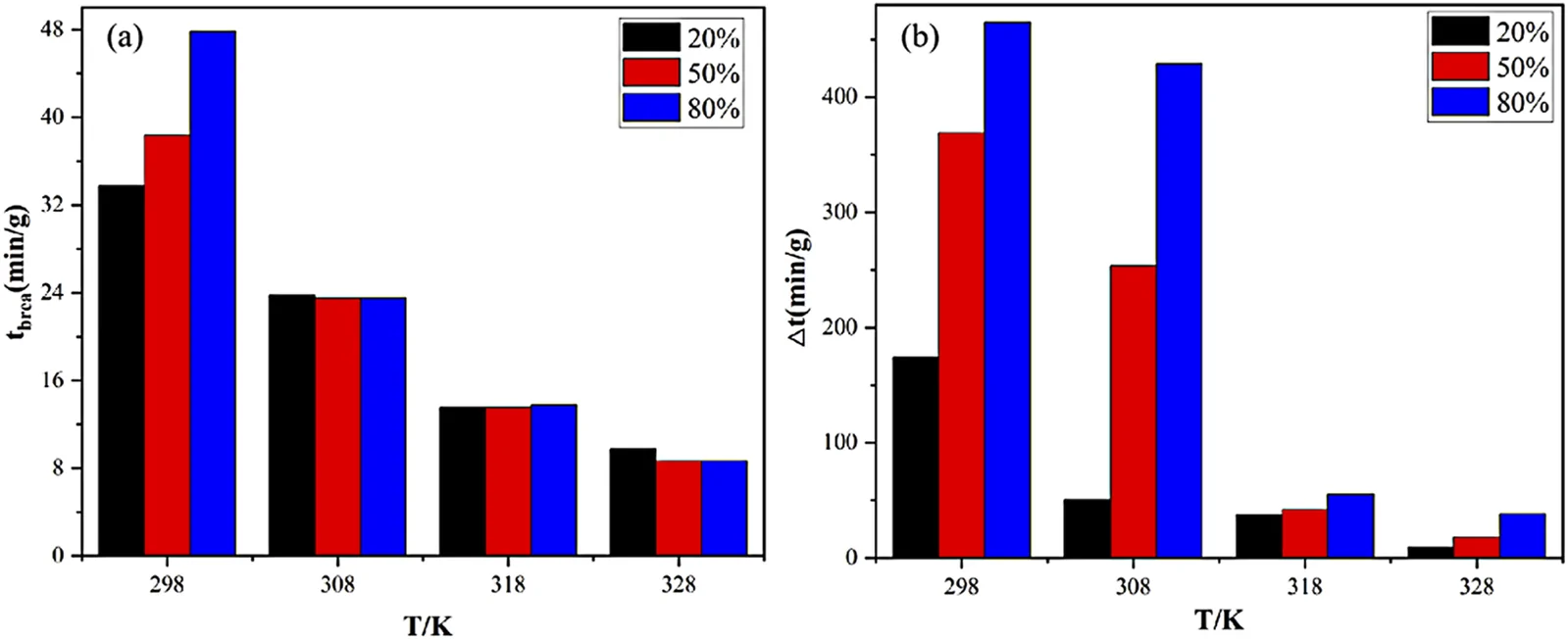
Variation of breakthrough time (a) and mass transfer time (b) at three RHs on GAC at four temperatures (298.15, 308.15, 318.15 and 328.15 K).

Breakthrough time and mass transfer are two crucial parameters for evaluating the dynamic performance. Given the unique shape of breakthrough curves of water vapor, especially at 298.15 K, calculating the mass transfer zone, which is widely regarded as an important indicator of the adsorption capacity of adsorbates,^17^ is challenging. To quantitatively assess the dynamic adsorption behavior, the mass transfer time (Δ*t*) was calculated as the interval between the breakthrough time (*t*_brea_) and the saturation time (*t*_sat_), based on the experimental data in Fig. 3. These parameters, tabulated in Table S3, were employed to characterize the mass transfer kinetics of water vapor. The variations in *t*_brea_ and Δ*t* under different relative humidity levels and temperatures were comprehensively illustrated in Fig. 4.

As shown in Fig. 4(a), it could be observed that the breakthrough time of water vapor on GAC at temperatures ranging from 308.15 K to 328.15 K remained constant across different relative humidity levels. These findings contrasted with previous reports on VOCs adsorption,^18^ wherein breakthrough time decreased with increasing VOC concentration. This divergence likely stemmed from the distinct equilibrium adsorption mechanisms governing water vapor *versus* VOCs, as elucidated in our prior work.^19^ Specifically, adsorption initiates with water molecules interacting with surface functional groups, followed by the progressive formation of water clusters, and ultimately culminates in micropore filling at elevated relative pressures. Prior to breakthrough, water vapor was predominantly adsorbed onto GAC through hydrogen bonding with surface acidic groups. Consequently, variations in relative humidity exerted a negligible impact on the breakthrough time. While notably, and in particular, it was evident that for granular activated carbon (GAC) at 298.15 K, the breakthrough time of water vapor exhibited an increase as the relative humidity rose. Drawing upon the equilibrium adsorption findings from our prior research,^19^ it could be observed that the adsorption capacity of water vapor on GAC at 298.15 K demonstrated a significant enhancement when compared to temperatures ranging from 308.15 K to 328.15 K. This was characterized by a more pronounced Type V isotherm profile. At 298.15 K, with higher relative humidity, water clusters were easier formed on the adsorbent surfaces, followed by capillary condensation to form a water film in the micropores.^20^ Therefore, the equilibrium adsorption capacity of GAC at 298.15 K exhibited a more marked increase in tandem with rising relative pressure, ultimately resulting in a substantially higher breakthrough capacity under elevated relative pressure conditions.

Regarding the mass transfer time (Δ*t*) (as shown in Fig. 4(b)), it could be observed that mass transfer time (Δ*t* = *t*_sat_ − *t*_brea_) increased with the rise of RH. This indicated that the dynamic adsorption rate decreased as RH increases, consistent with the trends observed in the adsorption kinetics of water vapor in our previous study.^14^ As the relative pressure of water vapor increased, the reduction in effective pore width and micropore filling lead to the formation of barriers to surface diffusion into the porous structure. Consequently, the dynamic adsorption rate continuously decreased with relative pressure and surface coverage, leading to wider mass transfer zone.

#### 3.1.2 Effect of temperature

**Fig. 5:**
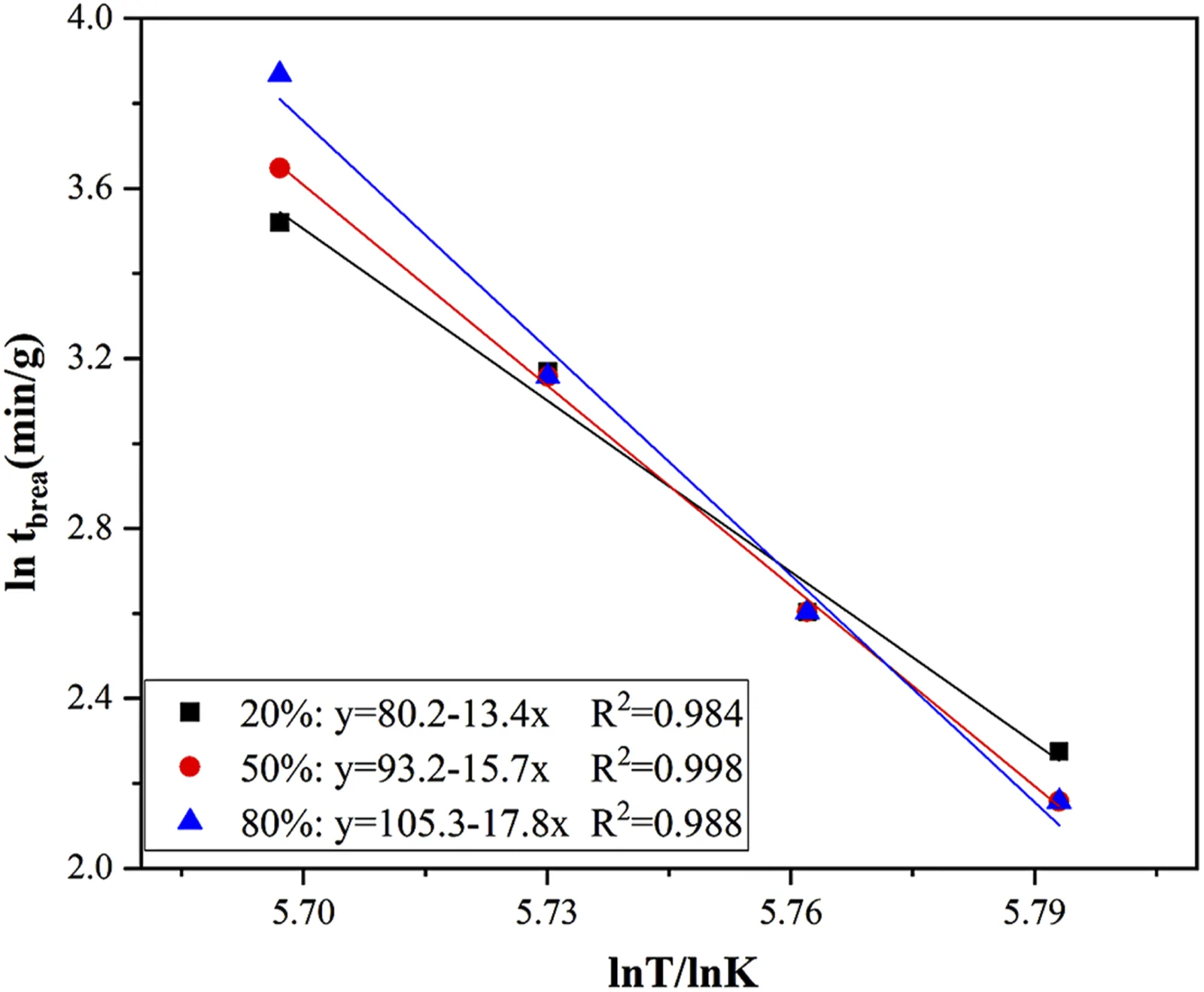
Effect of temperature on the breakthrough time of water vapor at three RHs (20%, 50% and 80%) on GAC.

As illustrated in Fig. 4, it could be observed that both the breakthrough time and the mass transfer time decreased as temperature rose. This indicated that the breakthrough adsorption capacity and the mass transfer resistance declined with increasing temperature. Regarding the breakthrough time, we further analyzed the relationship between the breakthrough time of water vapor and temperature, as presented in Fig. 5. It was evident that the breakthrough time exhibited a negative logarithmic correlation with temperature. Adsorption equilibrium served as a crucial factor influencing dynamic adsorption.^21^ Consequently, the dynamic adsorption capacity tended to follow a similar trend to that of the equilibrium adsorption capacity.^22^ At higher temperatures, it became more challenging for water molecules to form clusters. Additionally, water molecules required a higher potential energy to remain within the micropores.^23^ As a result, the breakthrough adsorption capacity of water vapor decreased with increasing temperature.

Turning to the mass transfer, a higher temperature provided more energy for the formation of hydrogen bonds, thereby resulting in a higher kinetic adsorption rate.^14^ Moreover, an increase in temperature led to an enhancement in the surface diffusivity of water vapor. Consequently, the mass transfer time decreased with the elevation of temperature. In addition, under conditions of high temperature, where surface coverage was minimal, water vapor molecules could more readily diffuse into the interior surfaces of the adsorbent. This facilitated a higher adsorption rate and, consequently, resulted in steeper slopes of the breakthrough curves. While notably in Fig. 3, for water vapor with relative humidity levels ranging from 50% to 80% at 298.15 K, it is clear that a plateau region was observed in the breakthrough curves of water vapor, consistent with the findings reported in the literature.^11,24^ This plateau likely corresponded to the growth of water clusters on primary adsorption sites and the subsequent formation of interconnecting bridges between these clusters. The duration of the plateau region correlated strongly with the time required for complete micropore filling by water clusters.^14^ Consistent with our prior findings,^19^ adsorption capacities at 298.15 K under high relative humidity (50% and 80%) were significantly elevated. This promoted the formation of a greater number of water clusters within the micropores, thereby resulting in a more pronounced plateau in the breakthrough curves. We attributed this anomalous extension of the water breakthrough duration at 298.15 K to enhanced cluster formation and micropore filling under low-temperature conditions, where reduced thermal motion favors water aggregation in confined nanopores.

### 3.2 Effect of water vapor on dynamic adsorption of acetone

#### 3.2.1 Dynamic adsorption performance of acetone during co-adsorption with water vapor

**Fig. 6:**
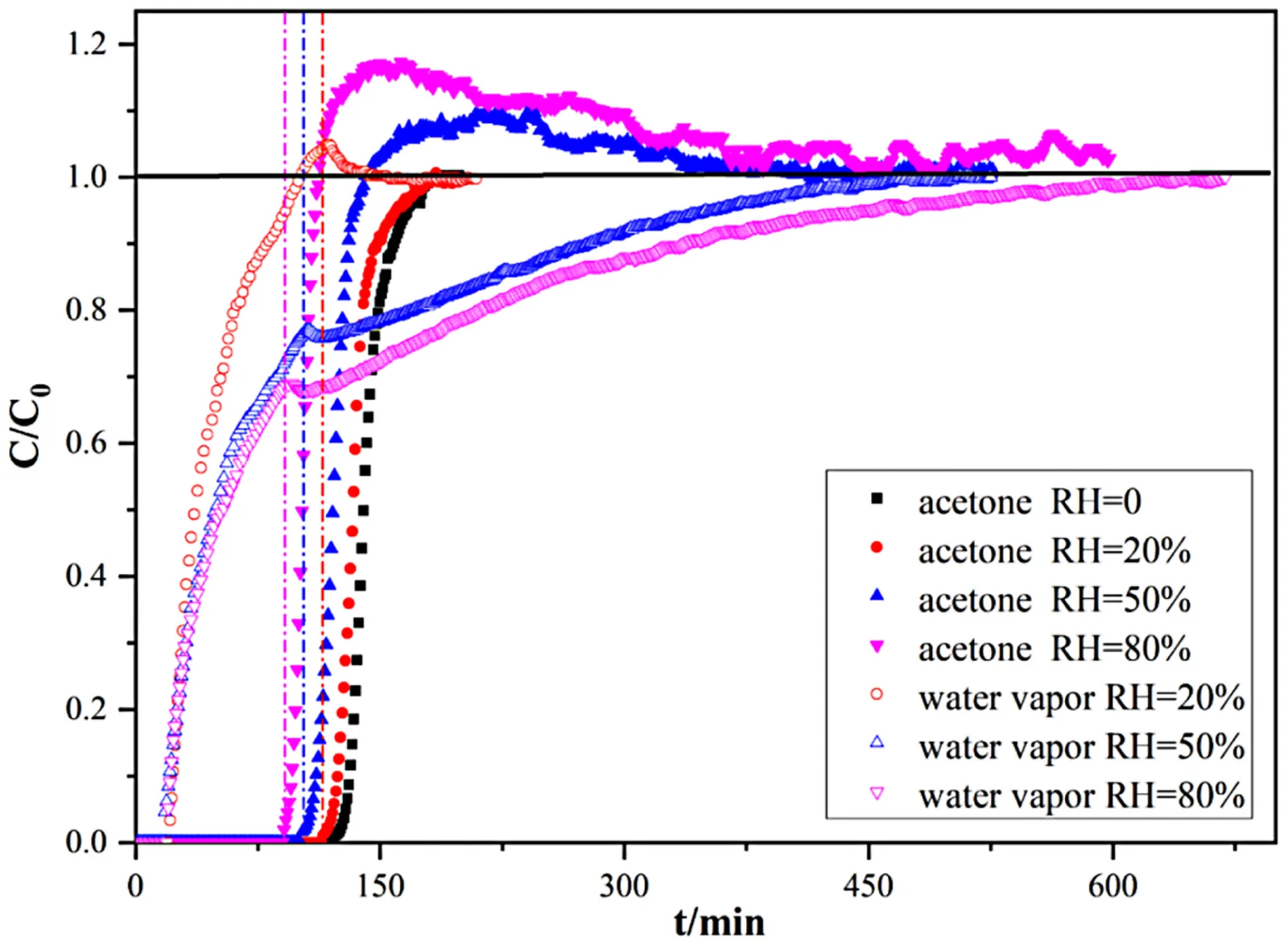
Breakthrough curves of acetone (10 mg L^−1^) on GAC at different RHs.

Building on the dynamic adsorption characteristics of water vapor, the co-adsorption behavior of acetone and water vapor was further investigated to elucidate the impact of humidity on VOC recovery. Fig. 6 presented the breakthrough curves of acetone (*C*_0_ = 10 mg L^−1^) on GAC under varying relative humidity (RH) levels at 298 K. The introduction of water vapor into the feed stream markedly compromised the dynamic adsorption capacity of acetone, as evidenced by a reduction in breakthrough capacity and the displacement of adsorbed acetone. This inhibitory effect was primarily attributed to the preferential occupation of active sites by water molecules through hydrogen bonding, which restricted the accessibility of acetone to the GAC surface.^25–27^

Furthermore, a distinct dual roll-up phenomenon was observed in the acetone-water co-adsorption system, as shown in Fig. 6. This behavior was attributed to the comparable adsorption affinities of acetone and water vapor for the GAC surface, signifying intense competition during the co-adsorption process. Notably, according to the height and integrated area of the roll-ups in the breakthrough curves of acetone in Table 1, the magnitude of the acetone roll-up effect intensified with increasing relative humidity (RH). This trend could be explained by analyzing the water vapor breakthrough profiles: as RH rose, the roll-up features in the water vapor curves diminished and eventually vanished, reflecting a marked enhancement in the competitive adsorption capacity of water vapor. At elevated RH, an increased number of water clusters with diameters approximating the micropore size were formed, thereby strengthening their adsorption affinity. This was particularly evident at 80% RH,^28^ where the superior competitiveness of water vapor led to more pronounced displacement of acetone, resulting in the exacerbated roll-up effect observed in Fig. 6.

**Table 1:** The height and area of the roll-ups for breakthrough curves of acetone

RH/%	Integrated area of roll-ups	Height of roll-ups
20	0	1.00
50	12.15	1.10
80	27.47	1.17

Regarding the roll-up phenomena in the water vapor breakthrough curves, the magnitude of the roll-up was considerably smaller than that observed for acetone, signifying a bidirectional competitive displacement interaction between the two components. Bidirectional competitive displacement refers to two concurrent processes: (1) water molecules displaced adsorbed acetone from high-affinity micropore sites *via* hydrogen bonding with oxygenated functional groups; (2) acetone molecules occupied pore channels, reducing pore blockage and accelerating water breakthrough. This phenomenon suggested that while water vapor occupied a portion of the micropores, rendering them inaccessible to acetone, the acetone molecules were capable of displacing weakly bound water molecules. These displaced species were primarily associated with outer-layer multilayer water and capillary-condensed water, which were less energetically stable than those involved in primary adsorption.^29^ Notably, the displacement effect of water molecules on acetone was more pronounced than that of acetone on water. As listed in Table S1, the dynamic diameter of a water molecule is 0.343 nm, whereas a water pentamer (cluster of five) measures approximately 0.7 nm,^30–32^ closely matching the micropore dimensions of GAC. According to Polanyi's potential theory, adsorbates exhibited stronger affinity for pores when their molecular size aligns with the pore diameter.^33^ Consequently, water clusters possessed a higher adsorption potential for GAC,^34^ particularly at 80% RH where capillary condensation dominated.^28^ This enhanced affinity explained why the roll-up magnitude of water vapor diminishes with increasing RH.

**Fig. 7:**
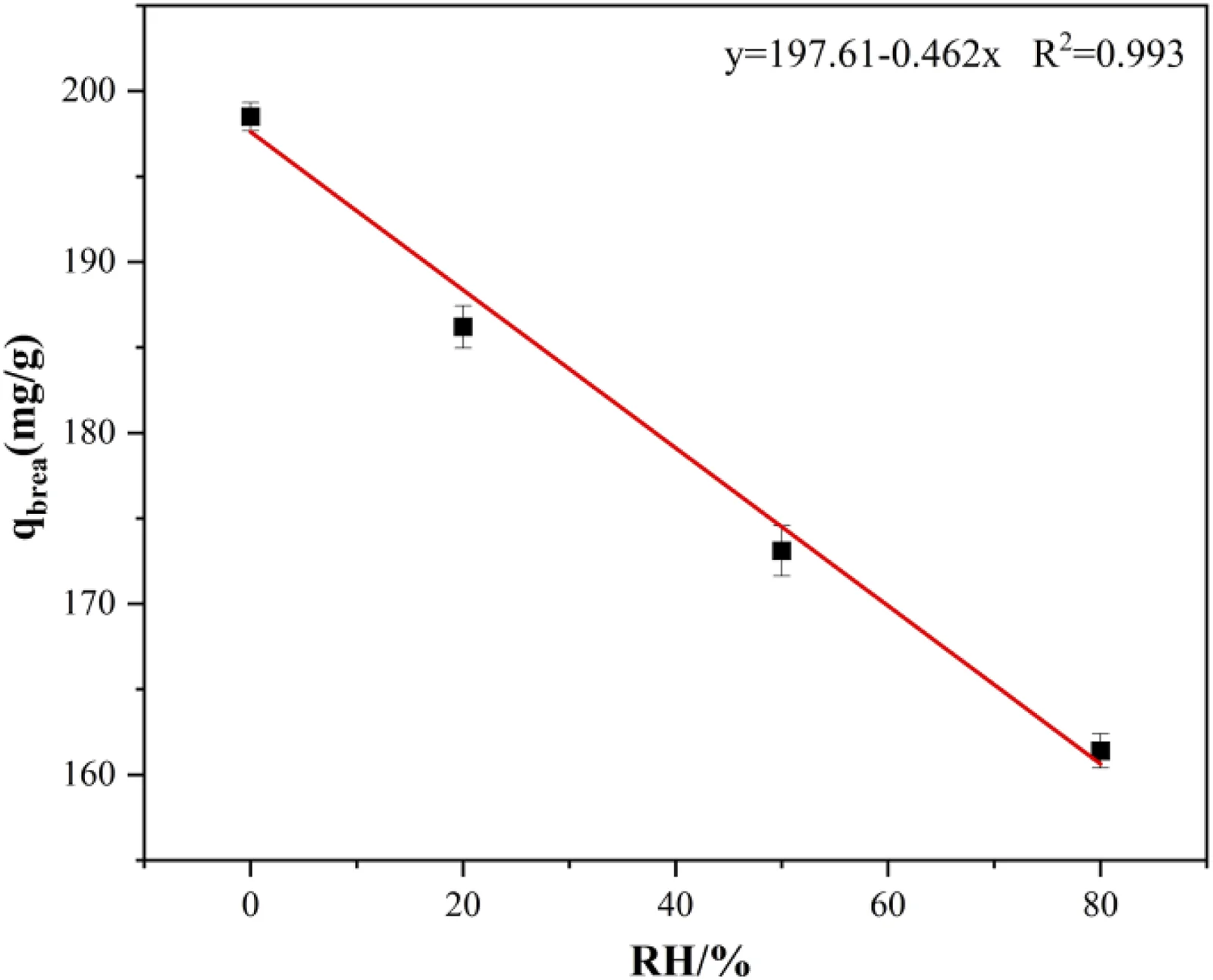
Breakthrough adsorption capacity of acetone *versus* RH on GAC.

Furthermore, based on the breakthrough (*t*_brea_) and saturation (*t*_sat_) times listed in Table 1, the mass transfer time (Δ*t* = *t*_sat_ − *t*_brea_) were calculated to characterize the dynamic adsorption of acetone. As shown in Table 2, both *t*_brea_ and Δ*t* decreased significantly with increasing relative humidity. This trend was further corroborated by the breakthrough capacities presented in Fig. 7, which exhibited a distinct negative linear correlation with RH, indicating that higher humidity severely impaired the adsorption efficiency of GAC for acetone. Correlating these results with Fig. 3, 4 and 6, it revealed that the adsorption capacity of water vapor increased with rising relative humidity, leading to the occupation of an increasing number of active sites. Consequently, the breakthrough time of acetone shortened significantly at higher RH. At low RH, the decline in acetone breakthrough capacity was primarily governed by competitive adsorption between water molecules and acetone for surface functional groups. However, as RH continued to rise, the adsorption capacity of acetone further deteriorated. This is attributed to the micropore filling and capillary condensation of water vapor, which physically blocked the micropores and reduced the available adsorption volume for acetone.^20^

**Table 2:** Breakthrough time (*t*_brea_) and mass transfer time (Δ*t*) of acetone adsorption on GAC at different relative humidity

RH/%	*t*_brea_/min	*t*_sat_/min	Δ*t*/min
0	121.9	185.5	63.6
20	114.9	176.5	61.6
50	99.1	138.8	39.7
80	87.2	110.9	23.8

Regarding the mass transfer time (Δ*t*), which is inversely related to the diffusion rate, they decreased linearly with increasing RH, indicating enhanced diffusivity. As evidenced by the data in Table 2, this acceleration was attributed to the displacement of acetone by water vapor. At higher RH, water molecules saturated the primary adsorption sites, leaving fewer available sites for acetone. This reduction in available adsorption sites weakened the overall adsorption affinity for acetone, thereby facilitating its mass transfer and resulting in a shorter Δ*t*.

**Fig. 8:**
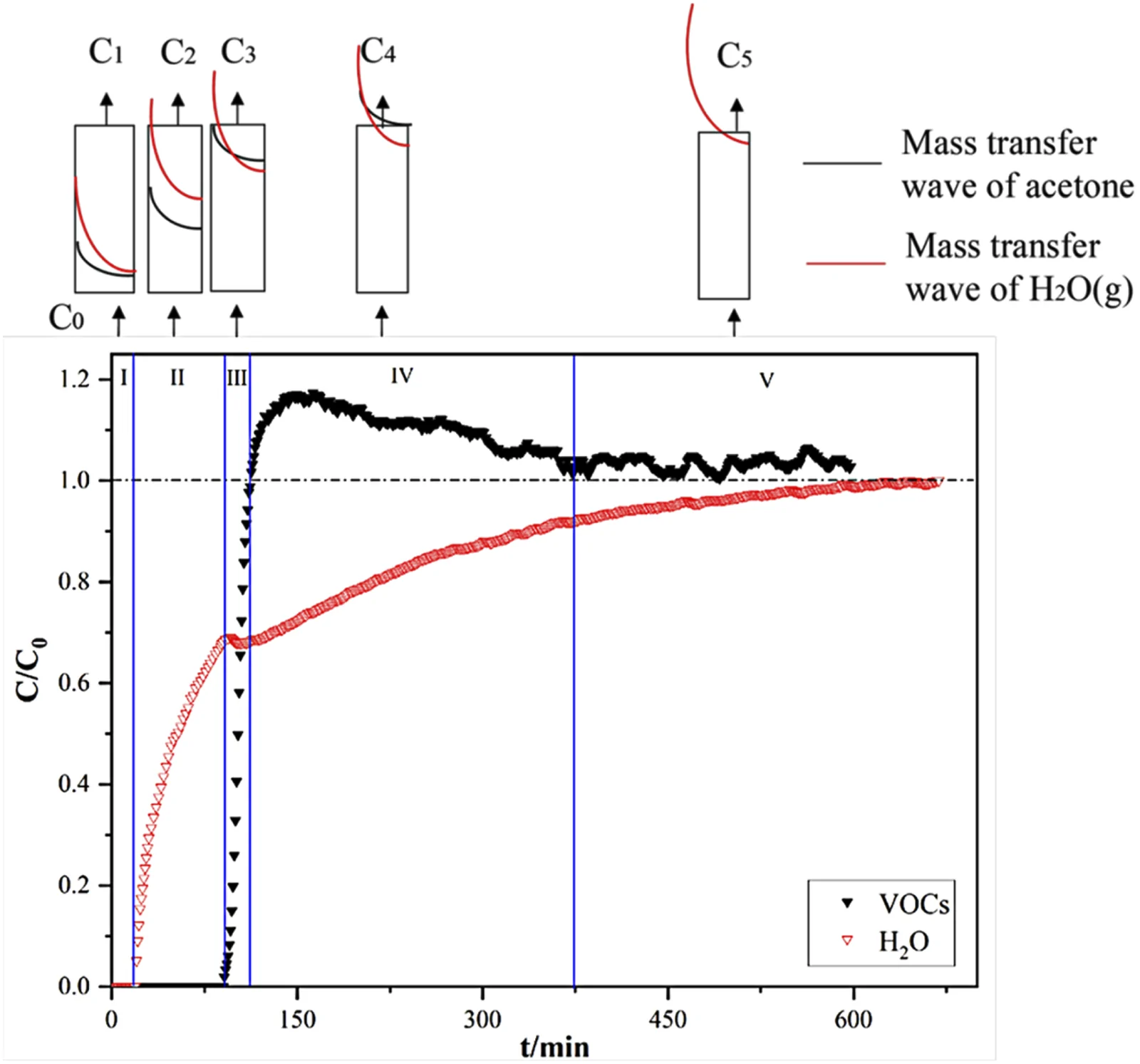
Dynamic co-adsorption scheme for acetone and H_2_O on GAC.

To conduct an in-depth analysis of the competitive dynamic adsorption between acetone and water vapor, we specifically focused on the co-adsorption system with water vapor at a relative humidity of 80%. Subsequently, we plotted the breakthrough curves along with the corresponding movement of the mass transfer wave, as presented in Fig. 8, dividing the dynamic process into five distinct stages. The mass transfer waves for acetone and water vapor were constructed based on their respective adsorption isotherms, which are classified as favorable and unfavorable types, respectively.

In Stage I, despite the abundance of available adsorption sites, competitive adsorption between acetone and water vapor was evident. Compared to the single-component water vapor adsorption (Fig. 3), the breakthrough time of water vapor (RH = 80% at 298.15 K) decreased dramatically by 88%, plummeting from 167 min to merely 20 min. This drastic reduction was attributed to two synergistic effects. On the one hand, adsorbed acetone occupied pore channels, creating diffusion resistance for incoming water molecules. When acetone was present in the feed gas, it displaced from micropores into larger pores during competitive adsorption, relieving pore blockage and reducing water's diffusion path length. On the other hand, acetone weakened the hydrogen bonding network between water and GAC surfaces, promoting faster water cluster formation and transport in pores. Together, these effects led to the dramatic acceleration of water breakthrough in binary systems. In Stage II, water vapor began to break through, accompanied by the emergence of a roll-up phenomenon in its breakthrough curve. This behavior was a direct manifestation of the intense competition between acetone and water vapor for adsorption sites on the GAC. Once water vapor broke through, the initially adsorbed water molecules act as secondary adsorption sites, promoting further adsorption and subsequent cluster formation. Concurrently, the expanding mass transfer zone of water vapor created opportunities for acetone to displace unstable, weakly bound water molecules—primarily outer-layer multilayer water and capillary-condensed water.^20,29^ However, at the acetone breakthrough point, only a marginal roll-up effect was observed in the water vapor breakthrough curve. This was attributed to the occupation of available micropores by water vapor, which blocked access for acetone molecules. Consequently, the breakthrough time of acetone (10 mg L^−1^) decreased from 122 min to 87 min, defining Stage III. In this stage, the onset of acetone breakthrough slowed the mass transfer rate of water vapor due to the intensifying competition for adsorption sites. Interestingly in the stage IV, a roll-up phenomenon was also observed in the breakthrough curves of acetone, where the outlet concentration rapidly surpassed the inlet level, peaking at approximately 1.2 times of inlet concentration. This behavior was attributed to the superior adsorption potential of water clusters for GAC at elevated RH, which allowed them to monopolize adsorption sites and displace adsorbed acetone molecules.^35^ As illustrated in Fig. 8, the mass transfer wave of water vapor was significantly broader and slower than that of acetone. Consequently, the trailing edge of the water vapor wave overtook the acetone wave, creating an obvious displacement event.^36^ Finally, in Stage V, acetone reached saturation, and water vapor continued to adsorb slowly onto the remaining available sites.

#### 3.2.2 Theoretical calculation of acetone-water co-adsorption

To further corroborate the influence of water on the adsorption of acetone, the adsorption heats, adsorption energy, radial distribution function (RDF) and mean square displacement (MSD) were meticulously computed based on the model construction diagram illustrated in Fig. 2. The results of adsorption heats showed that the average adsorption heat of C_3_H_6_O was 10.443 kcal mol^−1^, while that of H_2_O was 8.753 kcal mol^−1^. However, the maximum adsorption heat of H_2_O reached 22.192 kcal mol^−1^, which was higher than that of C_3_H_6_O, 19.892 kcal mol^−1^. This result revealed a distinct local enhancement effect for water molecules. Unlike acetone, water could access exceptionally strong adsorption sites *via* cooperative interactions within specific microenvironments.

GCMC simulations corroborated this mechanism, demonstrating a clear tendency for water molecules to form small hydrogen-bonded clusters. Structural analysis of the configurations obtained after 10 000 and 100 000 GCMC steps—using the O_water_–O_water_ distance as a criterion—illustrates this evolution. At a cutoff of 3.5 Å, the 10 000-step structure primarily consisted of isolated molecules and dimers, with no clusters larger than two water molecules. In contrast, the 100 000-step structure exhibited a marked increased in water–water contacts, with approximately 69% of adsorbed water molecules participating in aggregates. Several stable clusters containing 3–6 water molecules were observed, a trend that remained consistent even when a more conservative cutoff of 3.2 Å was applied.

**Fig. 9:**
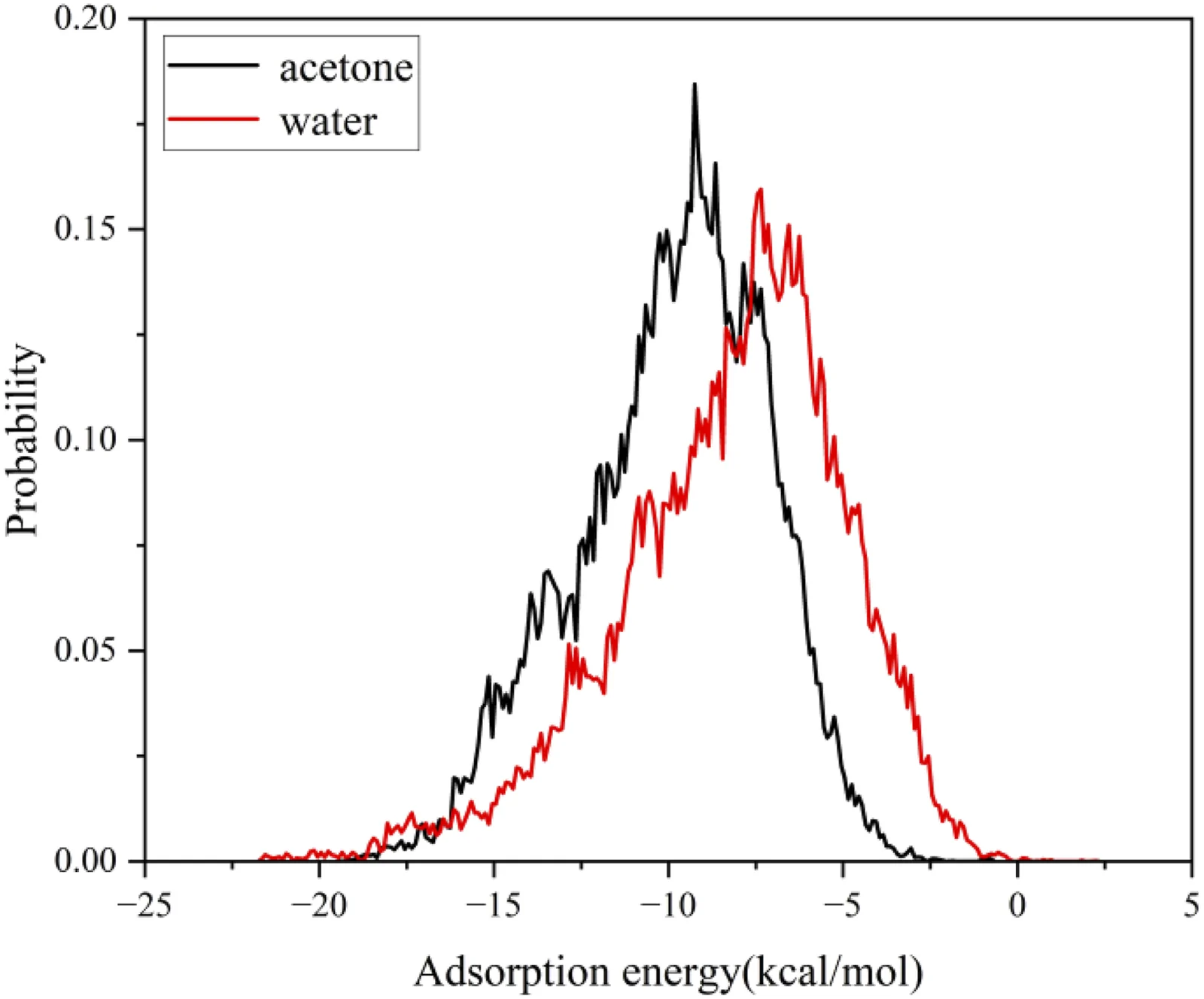
Adsorption energy distribution of acetone and water configurations in the co-adsorption system.

As shown in Fig. 9, the adsorption energy distribution of the co-adsorption system further supported this finding. Although water molecules didn't exhibit a higher average adsorption strength across all configurations, specific oxygen-containing functional groups facilitated the formation of strong local adsorption sites. Here, water–water cooperative interactions and extended hydrogen-bond networks synergistically enhanced the local adsorption affinity. This hydrogen-bond cooperativity provided a robust theoretical basis for the formation of stable water-cluster adsorption configurations on oxygenated carbon surfaces.

**Fig. 10:**
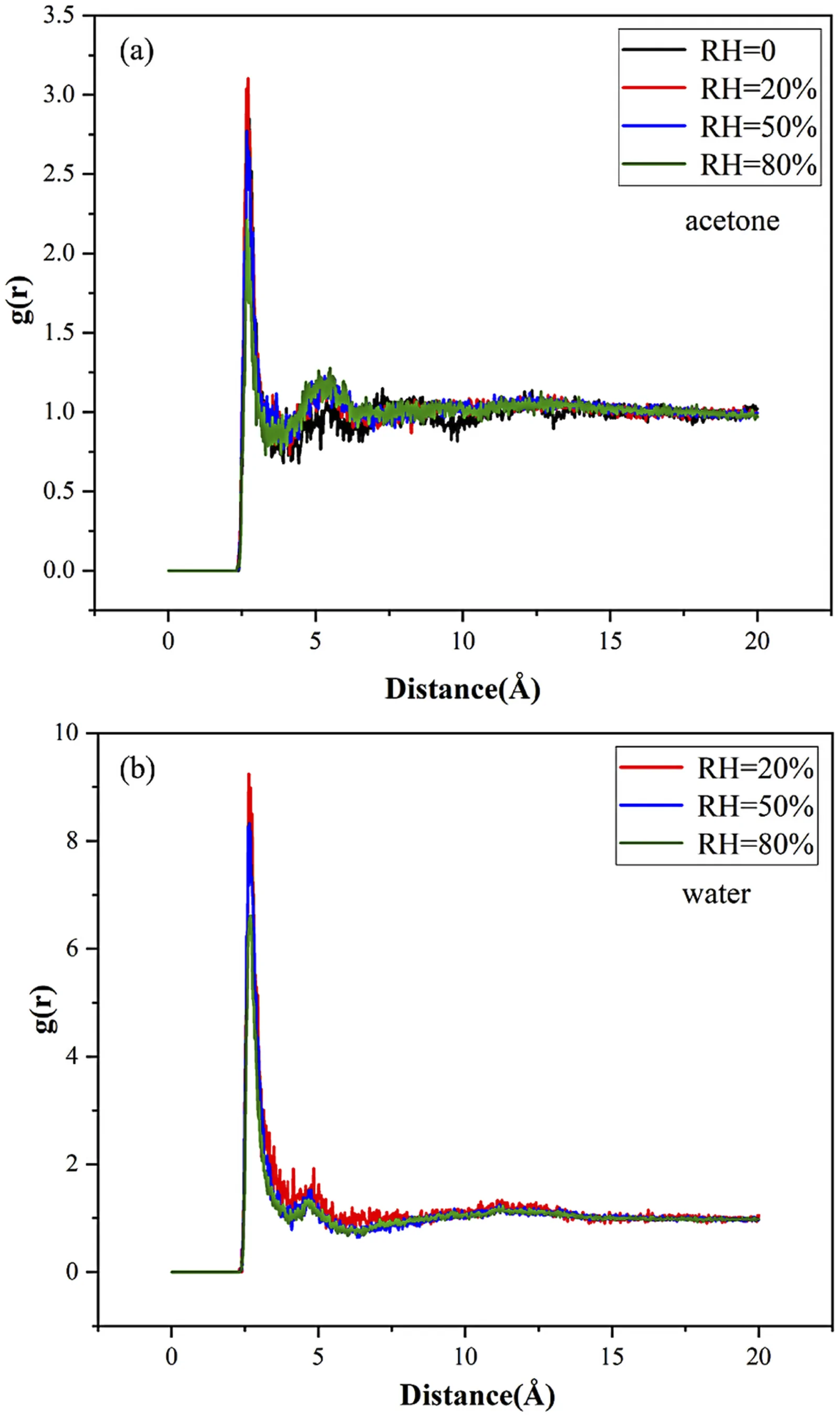
The radial distribution function results of acetone (a) and water (b) molecules on GAC during their co-adsorption.

Furthermore, the RDF served as a pivotal tool for assessing the spatial distribution of acetone or water molecules in the vicinity of adsorption sites on GAC.^17^ Specifically, the RDF results, denoted as *g*(*r*), which described the variation in guest molecule density as a function of distance (*r*) from the adsorption sites, were presented in Fig. 10. As shown, the RDF analysis revealed distinct density peaks for acetone and water molecules. Acetone exhibited two prominent density peaks at *r* ≈ 2.7 Å and 5.5 Å, while water demonstrated density peaks at *r* ≈ 2.7 Å and 4.7 Å. At *r* of 2.7 Å, the interplay between acetone and water molecules on GAC surface were obvious. It could be further noted in Fig. 10 that the change trend of RDF curve of acetone was basically the same, and it was little affected by changes in relative humidity.^21^ When the relative humidity increased from 0% to 80%, the minimum distance between the central atom of acetone and the surface of GAC was basically unchanged, which indicated that the presence of water molecules had little effect on the adsorption position of acetone. From the shape of the RDF curve, it could be seen that the amounts of adsorbed acetone molecules increased gradually, which made the RDF of acetone molecules around GAC increase rapidly.

**Fig. 11:**
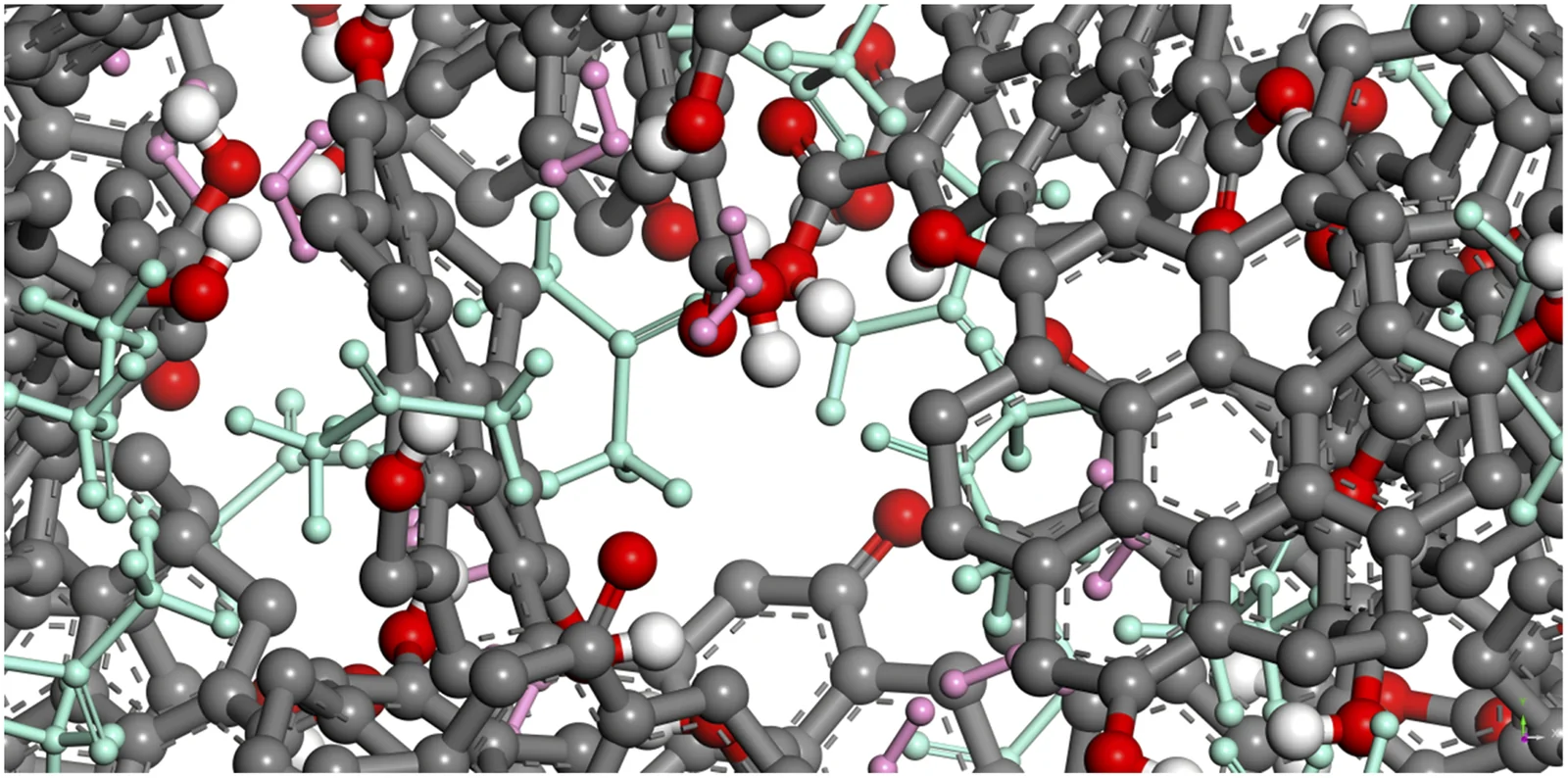
Co-adsorption snapshot of acetone and water near oxygen-containing functional groups.

In addition, the radial distribution function *g*(*r*) value of water molecules in Fig. 10 remained constant in both the single-component and binary-component adsorption systems. This indicated that the adsorption sites for water vapor were relatively unaffected by the presence of acetone. However, when acetone co-adsorbed with water vapor, its *g*(*r*) value at *r* ≈ 2.7 Å was lower compared to that in the single-component acetone adsorption system. Conversely, the *g*(*r*) value of acetone at *r* ≈ 5.5 Å increased in the presence of water vapor. This suggested that water vapor could occupied the adsorption sites of acetone at *r* ≈ 2.7 Å. According to Table S1, GAC contained four types of acidic groups: –C=O, –OH, –COOH, and –COOR. Among these, –OH and –COOH could function as either hydrogen bond donors (with the oxygen atom serving as a hydrogen bond acceptor), while –COOR and –C=O acted as hydrogen bond acceptors.^37^ Acetone consistently acted as a hydrogen bond acceptor. Therefore, the adsorption of acetone primarily involved interactions with –OH, –C=O and –COOH groups^38^ which could also formed hydrogen bond with water molecules, leading to fierce competition of these oxygenated functional groups for acetone and water vapor. This additional competition pathway further explained the reduction in acetone uptake under humid conditions. Based on the Fig. 11 of the nearest-neighbor distance analysis of the co-adsorption configuration, the average nearest contact distance between H_2_O molecules and oxygen-containing sites on the carbon substrate was approximately 3.00 Å, with the shortest contact distance being 1.64 Å. These contacts mainly occurred near –OH and –COOH, indicating that water molecules were preferentially adsorbed through hydrogen-bond donor/acceptor interactions with hydrophilic oxygen-containing functional groups. For C_3_H_6_O, the average nearest contact distance between the carbonyl oxygen of acetone and oxygen-containing sites was approximately 4.23 Å, with the shortest contact distance being 2.66 Å. The nearest adsorption sites were also mainly –OH and –COOH. The adsorption snapshots show that acetone tended to orient with its carbonyl oxygen pointing toward polar oxygen-containing groups, while the methyl groups were directed toward the carbon skeleton region. This indicated that acetone adsorption was governed by both polar interactions with oxygen-containing groups and dispersion interactions with the carbon framework.

To gain deeper insights into the interplay between acetone and water vapor during co-adsorption, mean square displacement (MSD) were meticulously computed and the results were presented in Fig. S1. According to eqn (2), we calculated the diffusion coefficient (*D*, Å^2^ ps^−1^) of adsorbates based on the slope of the MSD curve (*K*_MSD_).2
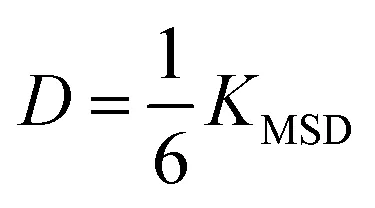


The diffusion coefficients (*D*) of acetone and water vapor were systematically analyzed, with the results presented in Table 3, with *R*^2^ higher than 0.995. The confidence intervals in Table 3 represented the fitting uncertainty of individual MSD curves rather than the standard deviation from independent replicate simulations. As listed in Table 3, the diffusion coefficients of acetone and water vapor were observed to increase in the binary adsorption system compared to the single-component system, and the diffusion coefficient of acetone presented a more significant rise. That is, both the diffusivity of acetone and water vapor within the pores of GAC enhanced. Consequently, acetone and water vapor displaced the other molecules mutually and the displacement of acetone by water vapor was more remarkable. These findings indicated that the inclusion of a small amount of water in the gas mixture could promote the diffusion of acetone. However, the concurrent increase in diffusion coefficients for both water vapor and acetone from the single-component to the binary adsorption system suggested that the coexistence of these two components was generally unfavorable for their respective adsorption processes.

**Table 3:** Diffusion coefficient (Å^2^ ps^−1^) of acetone in the co-adsorption system with different relative humidity

Adsorbates	H_2_O	Acetone
RH = 0	—	0.0505 ± 0.00769
RH = 20%	0.0461 ± 0.00439	0.1041 ± 0.00894
RH = 50%	0.1338 ± 0.00867	0.1410 ± 0.01023
RH = 80%	0.1483 ± 0.01036	0.1649 ± 0.01108

## 4 Conclusions

(1) Regarding the dynamic adsorption of water vapor, both the breakthrough adsorption capacity and the mass transfer resistance decreased with increasing temperature. While the breakthrough time of water vapor on GAC showed negligible dependence on relative humidity (RH) levels within the 308.15–328.15 K range, an exception was observed at 298.15 K, where the breakthrough time increased progressively with rising RH. Furthermore, for water vapor at 298.15 K with RH levels ranging from 50% to 80%, the breakthrough curves consistently exhibited a distinct plateau region. This phenomenon corresponds to the growth of water clusters on primary adsorption sites and the subsequent formation of interconnecting bridges between these clusters.

(2) In the co-adsorption system of acetone and water vapor at 298.15 K, the breakthrough profiles revealed a pronounced mutual inhibition between the two components, accompanied by a distinct bidirectional competitive displacement phenomenon. The amplified roll-up effect of acetone at elevated relative humidity (RH) was mechanistically linked to the formation of micropore-filling water clusters. These clusters possess an enhanced adsorption affinity, enabling them to effectively displace adsorbed acetone. Conversely, the diminished roll-up of water vapor at high RH was attributed to the size-exclusion effect: the physical dimensions of the water clusters closely match the GAC micropore diameter, allowing them to monopolize the adsorption sites and suppress any significant displacement by acetone.

(3) Based on molecular simulations, it concluded that while water had a lower average adsorption heat than acetone, its maximum adsorption heat exceeds that of acetone, reflecting a distinct local enhancement effect driven by cooperative hydrogen bonding in water clusters. RDF and nearest-neighbor distance analyses demonstrated that both acetone and water preferentially occupied limited hydrophilic –OH and –COOH sites, triggering fierce competition for oxygenated functional groups. And both species exhibited elevated diffusivity in binary systems, with acetone diffusion being significantly promoted by low-level water vapor. These findings clarified the molecular-level humidity effect on VOCs adsorption, providing a robust theoretical basis for optimizing fixed-bed adsorbers under humid operating conditions, and balancing trade-offs between adsorption capacity and mass transfer efficiency for practical VOCs control applications.

From a practical perspective, while water reduces the equilibrium adsorption capacity of acetone, the accelerated diffusion induced by low-level water vapor offers practical benefits for adsorption system operation. During adsorbent regeneration, introducing a small amount of water vapor can reduce the desorption activation energy and shorten the regeneration cycle, lowering energy consumption. For applications requiring short contact times, operating under moderate humidity conditions can improve acetone removal efficiency despite the reduced equilibrium capacity. This trade-off should be considered when optimizing fixed-bed operating parameters for specific industrial scenarios.

In this study, the observed bidirectional competitive displacement mechanism was primarily driven by the presence of polar oxygen-containing functional groups (–OH, –COOH) on GAC surfaces. For other carbonaceous adsorbents with similar surface chemistry (*e.g.*, activated carbon fibers, biochar), the same mechanism is expected to apply. For non-carbonaceous adsorbents with higher surface polarity (*e.g.*, zeolites, MOFs), water competition is anticipated to be even more severe due to stronger electrostatic interactions with hydrophilic frameworks. Further studies on diverse adsorbent-VOC pairs are warranted to validate the universality of the proposed mechanism across different porous materials. In addition, it should be noted that this study focused on a single GAC adsorbent and acetone as the model VOC. The quantitative relationships between RH, temperature, and adsorption performance may vary for other VOC species and adsorbents with different pore structures or surface chemistries. Future work will extend this study to a broader range of adsorbent-VOC systems and employ *in situ* characterization techniques to directly validate cluster formation in pores.

## Conflicts of interest

There are no conflicts to declare.

## Supplementary Material

RA-016-D6RA06366G-s001

## Data Availability

The data supporting the results of this study are available from the corresponding authors upon reasonable request. Supplementary information (SI): physicochemical properties of the VOCs and water are summarized in Table S1, while the characteristics of the GAC are listed in Table S2. Table S3 presents the breakthrough (*t*_brea_) and saturation (*t*_sat_) times of water vapor on GAC under varying relative humidities and temperatures. Additionally, the mean squared displacements (MSDs) of water and acetone during single-component and binary adsorption processes are illustrated in Fig. S1. See DOI: https://doi.org/10.1039/d6ra06366g.

## References

[cit1] Zeng J. Y., Zhang L. Y., Yao C. H., Xie T. T., Rao L. F., Lu H., Liu X., Wang Q., Lu S. (2020). Relationships between chemical elements of PM_2.5_ and O_3_ in Shanghai atmosphere based on the 1-year monitoring observation. J. Environ. Sci..

[cit2] Wang X. D., Yin S. S., Zhang R. Q., Yuan M. H., Ying Q. (2022). Assessment of summertime O_3_ formation and the O_3_-NOx-VOC sensitivity in Zhengzhou, China. Sci. Total Environ..

[cit3] Jia L., Shi Q., Xie S., Long C. (2018). Effect of pre-adsorbed water in hydrophobic polymeric resin on adsorption equilibrium and breakthrough of 1,2-dichloroethane. Adsorption.

[cit4] Xian S., Yu Y., Xiao J., Zhang Z., Xia Q., Wang H., Li Z. (2015). Competitive adsorption of water vapor with VOCs dichloroethane, ethyl acetate and benzene on MIL-101(Cr) in humid atmosphere. RSC Adv..

[cit5] Shi J., Han R., Lu S., Liu Q. (2021). A metal-OH group modification strategy to prepare highly-hydrophobic MIL-53-Al for efficient acetone capture under humid conditions. J. Environ. Sci..

[cit6] Laskar I. I., Hashisho Z., Phillips J. H., Anderson J. E., Nichols M. (2019). Competitive adsorption equilibrium modeling of volatile organic compound (VOC) and water vapor onto activated carbon. Sep. Purif. Technol..

[cit7] Jia L., Shi J., Long C., Lian F., Xing B. (2020). VOCs adsorption on activated carbon with initial water vapor contents: Adsorption mechanism and modified characteristic curves. Sci. Total Environ..

[cit8] Wang S., Hu J., Li L., Zuo S. (2024). Understanding the adsorption kinetics of acetone in humid activated carbons: perspectives from adsorption-breakthrough experiments and molecular simulations. ACS Omega.

[cit9] Fu J., Xu K., Ji Y., Wang X., Ma Y., Ostadhassan M., Pan Z., Wang D., Liu B., Ke Y., Sun M. (2025). Water vapor sorption behavior of shale organic matter with various types and maturation. J. Hydrol..

[cit10] Kim K. R., Lee M. S., Paek S., Yim S. P., Ahn D. H., Chung H. (2007). Adsorption tests of water vapor on synthetic zeolites for an atmospheric detritiation dryer. Radiat. Phys. Chem..

[cit11] Ribeiro R. P. P. L., Grande C. A., Rodrigues A. E. (2011). Adsorption of water vapor on carbon molecular sieve: thermal and electrothermal regeneration study. Ind. Eng. Chem. Res..

[cit12] Batur E., Kutluay S. (2022). Dynamic adsorption behavior of benzene, toluene, and xylene VOCs in single- and multi-component systems by activated carbon derived from defatted black cumin(Nigella sativa L.) biowaste. J. Environ. Chem. Eng..

[cit13] Baytar O., Şahin Ö., Horoz S., Kutluay S. (2020). High-performance gas-phase adsorption of benzene and toluene on activated carbon: response surface optimization, reusability, equilibrium, kinetic, and competitive adsorption studies. Environ. Sci. Pollut. Res..

[cit14] Jia L., Yao X., Ma J., Long C. (2017). Adsorption kinetics of water vapor on hypercrosslinked polymeric adsorbent and its comparison with carbonaceous adsorbents. Microporous Mesoporous Mater..

[cit15] Xie Y., Zheng C., Tang S., Song H., Kang K., Bai S. (2024). Adsorption of DMMP and water vapor on granular activated carbon: An experimental and molecular simulation study. Sep. Purif. Technol..

[cit16] Huang Y., Cheng Q., Wang Z., Liu S., Zou C., Guo J., Guo X. (2020). Competitive adsorption of benzene and water vapor on lignite-based activated carbon: experiment and molecular simulation study. Chem. Eng. J..

[cit17] Liu H., Yang B., Xue N. (2016). Enhanced adsorption of benzene vapor on granular activated carbon under humid conditions due to shifts in hydrophobicity and total micropore volume. J. Hazard. Mater..

[cit18] Wu J., Jia L., Wu L., Long C., Deng W., Zhang Q. (2016). Prediction of the breakthrough curves of VOC isothermal adsorption on hypercrosslinked polymeric adsorbents in a fixed bed. RSC Adv..

[cit19] Jia L., Niu B., Jing X., Wu Y. (2022). Equilibrium and hysteresis formation of water vapor adsorption on microporous adsorbents: Effect of adsorbent properties and temperature. J. Air Waste Manage. Assoc..

[cit20] Yao X., Liang X., Shi Y., Zhou J., Gong W., Bu Q. (2025). Investigation of the competitive adsorption mechanism between typical volatile organic compounds (VOCs) and water vapor in waste gas emissions from the fermentation industry. Sep. Purif. Technol..

[cit21] Shim W. G., Lee J. W., Moon H. (2006). Adsorption equilibrium and column dynamics of VOCs on MCM-48 depending on pelletizing pressure. Microporous Mesoporous Mater..

[cit22] Hartmann M., Bischof C. (1999). Mechanical stability of mesoporous molecular sieve MCM-48 studied by adsorption of benzene, n-heptane, and cyclohexane. J. Phys. Chem. B.

[cit23] Ohba T., Kaneko K. (2004). Cluster-growth-induced water adsorption in hydrophobic carbon nanopores. J. Phys. Chem. B.

[cit24] Shim W. G., Moon H., Lee J. W. (2006). Performance evaluation of wash-coated MCM-48 monolith for adsorption of volatile organic compounds and water vapors. Microporous Mesoporous Mater..

[cit25] Guo X., Li X., Gan G., Wang L., Fan S., Wang P., Tade M. O., Liu S. (2021). Functionalized activated carbon for competing adsorption of volatile organic compounds and water. ACS Appl. Mater. Interfaces.

[cit26] Cheng T., Li J., Ma X., Zhou L., Wu H. (2023). Behavior of VOCs competitive adsorption with water vapor in a slit-shaped phosphoric acid mesoporous activated carbon model. Sep. Purif. Technol..

[cit27] Zhu M., Zhou K., Sun X., Zhao Z., Tong Z., Zhao Z. (2017). Hydrophobic N-doped porous biocarbon from dopamine for high selective adsorption of p-Xylene under humid conditions. Chem. Eng. J..

[cit28] Águeda V. I., Crittenden B. D., Delgado J. A., Tennison S. R. (2011). Effect of channel geometry, degree of activation, relative humidity and temperature on the performance of binderless activated carbon monoliths in the removal of dichloromethane from air. Sep. Purif. Technol..

[cit29] Heinen A. W., Peters J. A., Bekkum H. V. (2000). Competitive adsorption of water and toluene on modified activated carbon supports. Appl. Catal., A.

[cit30] Ohba T., Kaneko K. (2011). Kinetically forbidden transformations of water molecular assemblies in hydrophobic micropores. Langmuir.

[cit31] Freeman J. J., Tomlinson J. B., Sing K. S. W., Theocharis C. R. (1995). Adsorption of nitrogen and water vapour by activated Nomex® chars. Carbon.

[cit32] Kaneko K., Hanzawa Y., Iiyama T., Kanda T., Suzuki T. (1999). Cluster-mediated water adsorption on carbon nanopores. Adsorption.

[cit33] Yang K., Xing B. (2010). Adsorption of organic compounds by carbon nanomaterials in aqueous phase: Polanyi theory and its application. Chem. Rev..

[cit34] Cheng T., Li J., Ma X., Zhou L., Wu H., Yang L. (2022). Alkylation modified pistachio shell-based biochar to promote the adsorption of VOCs in high humidity environment. Environ. Pollut..

[cit35] Zhang X., Lv X., Shi X., Yang Y., Yang Y. (2019). Enhanced hydrophobic UiO-66 (University of Oslo 66) metal-organic framework with high capacity and selectivity for toluene capture from high humid air. J. Colloid Interface Sci..

[cit36] Kim J. H., Ryu Y. K., Haam S., Lee C. H., Kim W. S. (2001). Adsorption and steam regeneration of n-hexane, MEK, and toluene on activated carbon fiber. Sep. Sci. Technol..

[cit37] Fletcher A. J., Uygur Y., Mark Thomas K. (2007). Role of surface functional groups in the adsorption kinetics of water vapor on microporous activated carbons. J. Phys. Chem. C.

[cit38] Liang X., Chi J., Yang Z. (2018). The influence of the functional group on activated carbon for acetone adsorption property by molecular simulation study. Microporous Mesoporous Mater..

