# Supplementary material for: Mechanistic insights into water vapor adsorption behavior and its impact on dynamic adsorption of acetone: experimental and molecular simulation study
Source: RSC Adv. 2026 Aug 27;16(42):50836–47. doi: 10.1039/d6ra06366g (PMC13519978; doi:10.1039/d6ra06366g)
Supplement: Supplementary file 1 [file RA-016-D6RA06366G-s001.pdf]

Supplementary Information (SI) for RSC Advances.
This journal is © The Royal Society of Chemistry 2026

Supplementary information

Mechanistic Insights into Water Vapor Adsorption Behavior and Its Impact on Dynamic Adsorption of Acetone: Experimental and Molecular Simulation Study

Lijuan Jia[1,*], Yue Guan[1], Yurun Tong[1], Yahan Cui[1], Tong Zhang[1], Kangbo Shen[1], Jinhao Zhang[1], Dayang Wu [1], Yiyang Guo[1]

[1]Shanxi Key Laboratory of Ecological Protection and Resources Utilization of Yuncheng Salt Lake, Department of Applied Chemistry, Yuncheng University, Yuncheng 044000, China

[*] Corresponding author

Shanxi Key Laboratory of Ecological Protection and Resources Utilization of Yuncheng Salt Lake, Department of Applied Chemistry, Yuncheng University, 1155 Fudan West Street, Yuncheng 044000, China. E-mail: jialijuan1203@163.com

Tel:0359-2610040

Table S1. Physic-chemical properties of VOCs[1] and water

VOCs	MW[a]	BP[b]/°C	P_e[c]/10^{-24} cm^3	V_m[d]/(cm^3/mol)	R[e]	Å[f] /nm
acetone	58.1	56.2	6.33	75.1	15.9	0.450
water	18.0	100.0	1.48	0.18	1.33	0.343

[a]Molecule weight [b]Boiling point [c]Polarizability [d]Molar volume

[e]Molar refraction [f]Molecular dynamic diameter

[g]Octanol-Water Partition Coefficient

Table S2. Selected properties of GAC[2]

Pore structure	value	Surface functional groups	value
S_{BET}(m^2/g)	2089.3	-OH (mmol/g)	0.984
V_{micro}(mL/g)	0.84	-C=O (mmol/g)	0.196
V_{meso}(mL/g)	0.78	-COOH (mmol/g)	0.517
Average pore diameter(nm)	2.70	-COOR (mmol/g)	0.004
		Total acidic (mmol/g)	1.701

Table S3. Breakthrough time (t_{brea}) and saturated time (t_{sat}) of water vapor at different relative humidity and temperatures on GAC

T/K	t_{brea}(min/g)			t_{sat}(min/g)		
	20%	50%	80%	20%	50%	80%
298	33.78	38.38	47.84	208.11	407.30	512.70
308	23.78	23.51	23.51	74.32	277.03	452.43
318	13.51	13.51	13.78	51.08	55.41	68.92
328	9.73	8.65	8.65	18.65	26.76	46.76

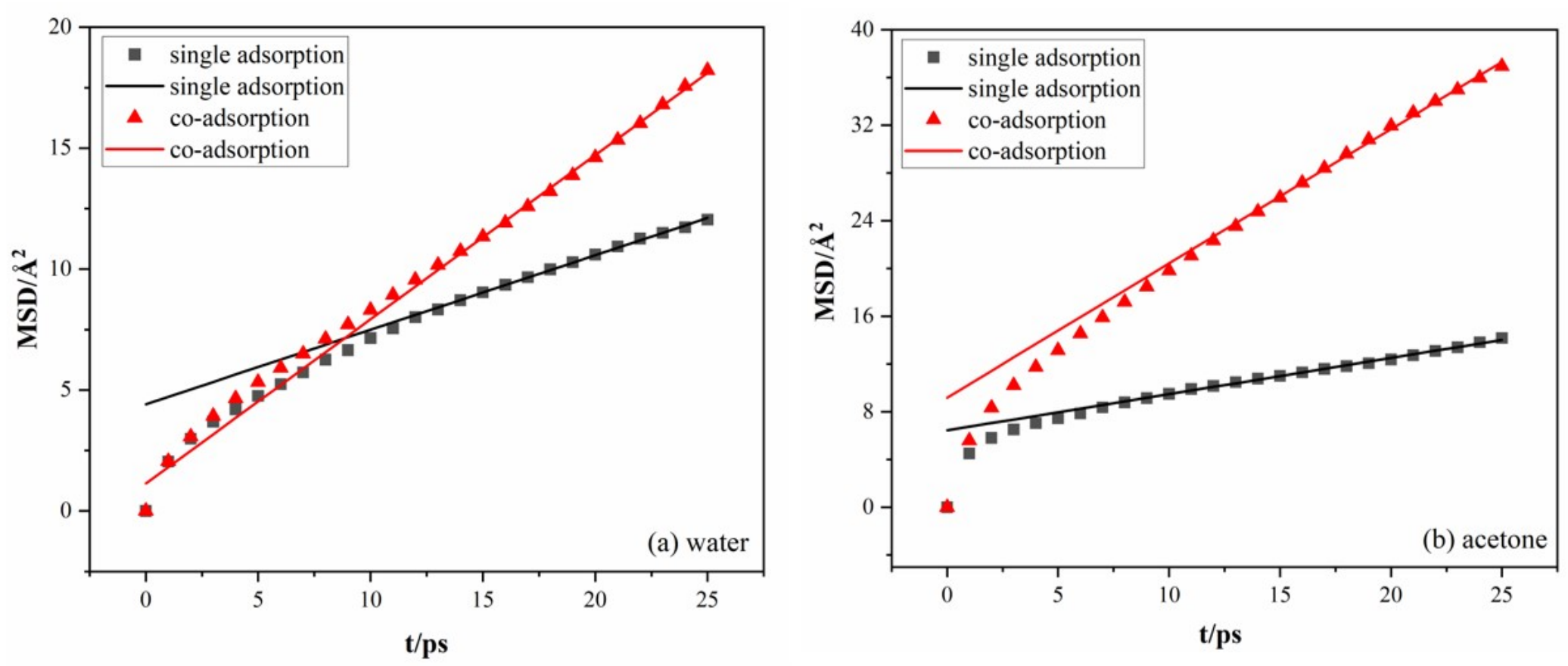

Figure S1. Mean square displacement(MSD) of water(a) and acetone(b) during single and binary adsorption

References

1 N. A. Khan, Z. Hasan, S. H. Jhung, *J. Hazard. Mater.*, 2013, **244-245**, 444-456.

DOI：10.1016/j.jhazmat.2012.11.011.

2 L. Jia, X. Yao, J. Ma and C. Long, *Micropor. Mesopor. Mater.,* 2017, **241**,178-184.

DOI:10.1016/j.micromeso.2016.12.028.
